# Health-Related Internet Use by Children and Adolescents: Systematic Review

**DOI:** 10.2196/jmir.7731

**Published:** 2018-04-03

**Authors:** Eunhee Park, Misol Kwon

**Affiliations:** ^1^ School of Nursing University at Buffalo Buffalo, NY United States

**Keywords:** Internet, child, adolescent, health information, health-related Internet use, eHealth

## Abstract

**Background:**

The internet is widely used by children and adolescents, who generally have a high level of competency with technology. Thus, the internet has become a great resource for supporting youth self-care and health-related services. However, few studies have explored adolescents’ internet use for health-related matters.

**Objective:**

The objective of this systematic literature review was to examine the phenomenon of children and adolescents’ health-related internet use and to identify gaps in the research.

**Methods:**

A total of 19 studies were selected from a search of major electronic databases: PubMed, Cumulative Index of Nursing and Allied Health Literature, and PsycINFO using the following search terms: “health-related internet use,” “eHealth,” “Internet use for health-related purpose,” “Web-based resource,” “health information seeking,” and “online resource,” combined with “child,” “adolescent,” “student,” “youth,” and “teen.” The children’s and adolescents’ ages were limited to 24 years and younger. The search was conducted from September 2015 to October 2017. The studies identified to contain youth (<24 years) health-related internet use were all published in peer-reviewed journals in the past 10 years; these studies examined general internet use seeking health care services, resources, information, or using the internet for health promotion and self-care. Studies were excluded if they explored the role of the internet as a modality for surveys, recruitment, or searching for relevant literature without specifically aiming to study participants’ health-related internet use; focused solely on quality assurance for specific websites; or were designed to test a specific internet-based intervention.

**Results:**

Interesting patterns in adolescents’ health-related internet use, such as seeking preventative health care and specific information about medical issues, were identified. Quantitative studies reported rates of the internet use and access among youth, and the purpose and patterns of health-related internet use among youth were identified. A major objective of health-related internet use is to gain information, but there are inconsistencies in adolescents’ perceptions of health-related internet use.

**Conclusions:**

This study’s findings provide important information on how youth seek information and related support systems for their health care on the internet. The conceptual and methodological limitations of the identified studies, such as the lack of a theoretical background and unrepresentative samples, are discussed, and gaps within the studies are identified for future research. This review also suggests important features for potential Web-based health interventions for children and adolescents.

## Introduction

The internet is widely used by children and adolescents, who generally exhibit a high level of competency with technology [1,2]. Its unique features and major benefits, such as highly engaging and motivating virtual components, as well as the portable, multitasking tools that give users easy and fast access to computers and mobile devices, mean that the internet has become a prevalent mode of communication and networking among youth [3,4]. Adolescents engage in many different activities on the internet, such as information searching, sharing personal information and artifacts, social media use, and recreational activities [5]; up to a quarter of their time is spent using multiple forms of media simultaneously, also known as multi-tasking [6]. As youth have a generally high level of access to the internet in their daily lives [7], it has become a major resource for them in supporting their self-care and health-related activities and services [8-10]. Although the internet is widely accessible and is well accepted by young people, there is as yet only a limited understanding of the patterns and characteristics of youth health-related internet use.

There are different patterns of the internet use by the various subgroups of this population depending on their developmental, gender, and social characteristics. As children progress to early adolescence, general internet usage increases and then levels off, presumably because of the heavier academic workload that teenagers must shoulder when they enter high school [11-13]. Similarly, research conducted on gender differences in internet use during adolescence is inconclusive [14]. Some studies have found boys (58%) to be more frequent users of the internet compared with girls (44%) [15], whereas other studies observed no significant gender difference in internet usage [16,17]. Children and adolescents also display notably different behavior in diverse regions of the globe depending on the local cultural, economic, and technological landscapes in their use of computers, mobile devices, and the internet. For example, a recent study from a cross-cultural context reported that the issue of internet addiction is not restricted to regions with high internet availability [18]. Data have shown that only 20% of African students reported spending an average of over 2 hours per day online compared with 42% and 40% of Chinese and US students, respectively [18]. However, despite the fact that access to the internet is much more limited than in either the United States or China, internet addiction is actually more prevalent in Africa [18].

The availability of high-quality health information can have a significant impact on the health outcomes of an individual. Health-related internet use is known to be associated with socioeconomic status, which is referred to as the digital divide [19]. Information obtained from interpersonal, online, or media sources facilitates the dissemination of new information, as well as influences how individuals shape their experience of health and illness [20]. This is true especially among young adults as they recognize social media as useful sources of information to supplement those received during their health care visits [21]. Online communities and social media are used to enhance access to valuable support networks, foster social inclusion, and facilitate peer-to-peer connections among adolescents with short-term or long-term diagnoses [21,22].

Young people have unique characteristics and can therefore pose special challenges for health promotion. During adolescence, teenagers undergo biological developments that involve physical, emotional, social, and pubertal maturation [23,24]. Due to these unique developmental characteristics, adolescence is also considered the most vulnerable period for engaging in various risky behaviors such as smoking, drugs, and sex [23]. However, adolescents also tend to form healthy habits and learn appropriate practices for their health concerns and management that can last for the rest of their lives [25]. Thus, youth is a critical period for the development of good health practices, highlighting the need to provide specific guidance for information and support related to their health and developmental milestones [26].

The internet offers many potential benefits for adolescent health promotion, including increasing the number of interventions for diverse topics related to the use of the internet among young people [27,28]. However, there is only a limited understanding of health-related internet use among children and adolescents. The purpose of this review is thus to conduct a systematic analysis of the research on this topic during the last 10 years and use the results to develop suggestions for important features that support effective Web-based health interventions for children and adolescents. The specific aims of this systematic review are as follows: (1) to describe the phenomenon of children and adolescents’ health-related internet use, (2) to identify benefits and barriers to health-related internet use for children and adolescents, and (3) to examine conceptual and methodological issues in the current literature.

## Methods

### Search Overview

**Figure 1:**
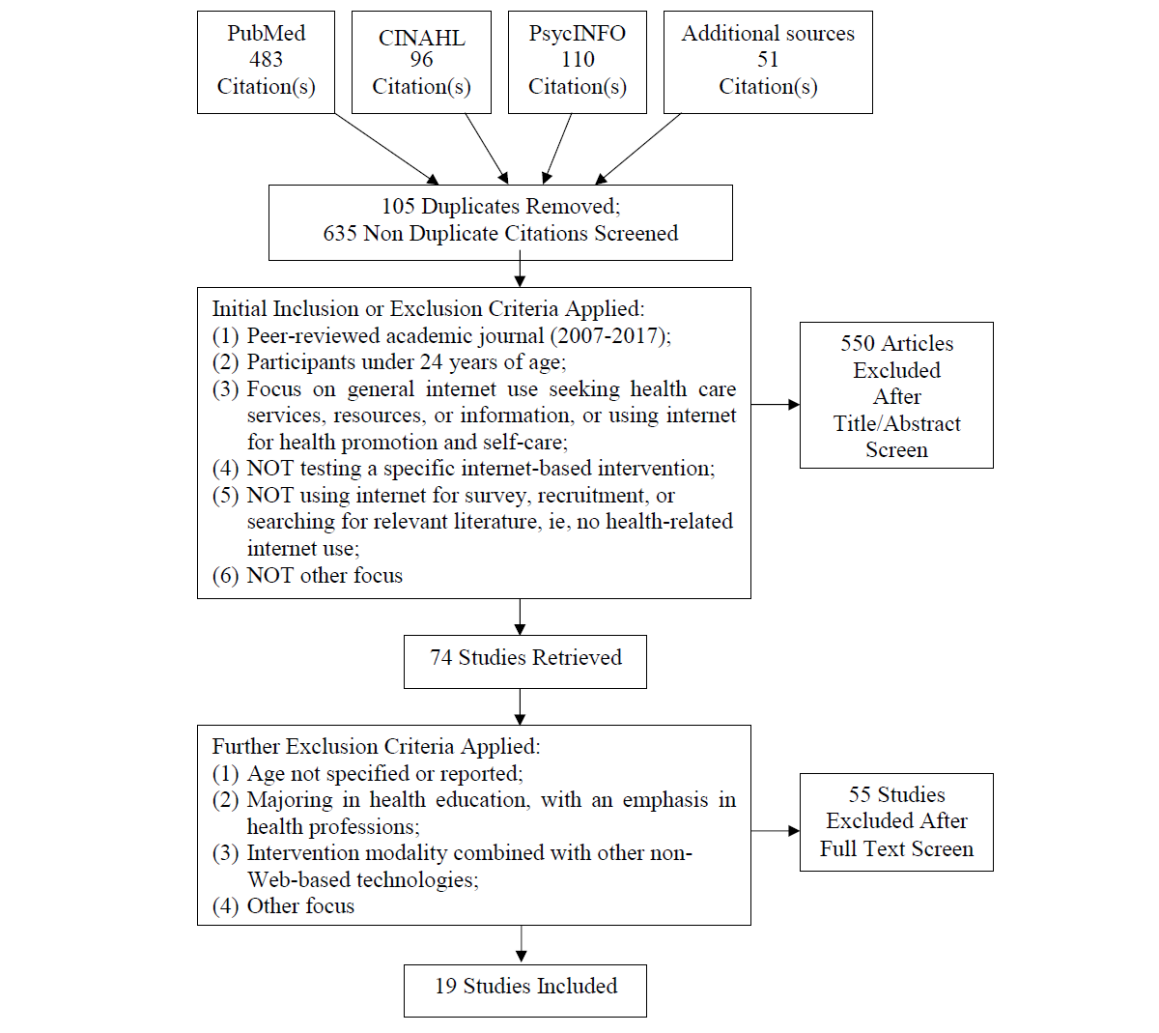
Flowchart of the literature search process. CINAHL: Cumulative Index of Nursing and AlliedHealth Literature.

The Preferred Reporting Items for Systematic Reviews and Meta-Analyses provides useful guidelines for systematic review studies [29]. This review is registered at PROSPERO (International Prospective Register of Systematic Reviews). On the basis of a careful consideration of the purposes of the study, inclusion and exclusion criteria are established to guide the subsequent search process, as shown in Figure 1.

### Search Strategy

Studies were selected from a search of three major electronic databases: PubMed, the Cumulative Index of Nursing and Allied Health Literature (CINAHL), and PsycINFO. An additional search was conducted using Google Scholar. Studies were also retrieved from the reference lists of the included studies. The search terms consisted of “health-related internet use,” “eHealth,” “internet use for health-related purpose,” “Web-based resource,” “online resource,” and “health information seeking,” combined with “child,” “adolescent,” “student,” “youth,” and “teen.” The studies were restricted to those concerning children and adolescents aged 24 years and under. The initial search was conducted from September 2015 to October 2017. Studies were included regardless of the location of the study to provide the broadest possible perspective of health-related internet use by young people. Adopting a global perspective was expected to enable us to examine a wide range of diverse phenomena, some of which could depend on the target population and where the study was conducted.

This study includes those who are up to 24 years to gain a comprehensive picture of health-related internet use among young people. Although there is no universal definition of adolescence, it is traditionally assumed to refer to youth from 12 to 18 years of age, with those in the age range of 18 to 24 years being considered late adolescents or young adults [30]. As there has been no previous systematic study of the health-related internet use of this population, our study was intentionally adjusted to include a broader age range and thus provide a deeper understanding of the unique characteristics of health-related internet use among these subpopulations (both younger and older adolescents) irrespective of location.

The initial search identified 740 studies. After the removal of 105 duplicates, the titles and abstracts of 635 studies were reviewed to determine whether they met the inclusion criteria, resulting in a list of 74 potentially relevant studies. The full texts of these studies were then retrieved for in-depth analysis by two independent reviewers to confirm both the inclusion and exclusion criteria listed below were met, which led to 55 studies being excluded. The remaining 19 studies were included (Figure 1).

**Inclusion criteria.**

Studies were included if

- they were published in a peer-reviewed academic journal from 2007 to 2017
- the study participants were under 24 years of age
- the studies examined general internet use seeking health care services, resources, or information or use of the internet for health promotion and self-care
- they were written in either English or Korean

**Exclusion criteria.**

Studies were excluded if

- the study participants were mixed with other populations aged 24 years or older
- the ages of the participants were not specified or reported
- the study participants were trained or were training to become professional health care providers (ie, physicians, nurses, or medical or nursing students)
- the intervention modality was combined with other non-Web-based technologies (such as telephones)
- the internet was simply a modality for conducting surveys or recruitment, or searching for relevant literature, rather than studying participants’ health-related internet use
- the study focused solely on quality assurance for specific websites;
- the study consisted of “gray literature” such as dissertations, papers or abstracts in conference proceedings, or editorials
- the study focused on testing a specific internet-based intervention

### Eligibility Criteria

The inclusion and exclusion criteria for studies are shown in Textboxes 1 and 2.

### Data Extraction, Analysis, and Synthesis

One of the authors initially reviewed the titles and abstracts based on the purpose of the study and the inclusion or exclusion criteria, after which two reviewers independently reviewed the full texts of the studies that were initially selected and coded them into an analysis table. The coding scheme was developed to help identify the components relevant to the study design and to address the first two research questions. The coding scheme included the year of publication, purpose of the study, country, number of participants, participants’ characteristics (eg, medical conditions and age), theoretical framework, main constructs, definition of health-related internet use provided, prevalence of health-related internet use, research design, sampling, data collection methods, instruments (including reliability and validity), data analysis, major findings, and study limitations. The coding also identified whether the findings of each of the quantitative studies indicated positive or negative perceptions about health-related internet use, as well as whether more than 50% of the participants had ever used the internet for health-related purposes. The qualitative and the quantitative studies that did not report these findings were coded as nonapplicable.

For the third research question, a coding table was created based on the guidelines suggested by the Agency for Healthcare Research and Quality criteria [29,31] that considered the research design, conceptual framework, sampling method, data collection method, instrument, analytic method, and threats to validity. The coding indicated whether the study utilized random, purposive, or convenience sampling; was quantitative or qualitative; where the data were collected, conducted at a single or multiple sites, and was an online survey, pen and pencil survey, interview, or focus group; and if the study utilized appropriate statistical analytic methods such as descriptive statistics, univariate regression and multivariate regression, or qualitative methods such as thematic analysis and content analysis. Potential threats to validity, such as self-report, a single site study, or self-selection bias were also identified and coded accordingly.

After coding tables were completed, the authors independently checked for discrepancies in the coded results to ensure accuracy. In the case of disagreement between authors, external review from experts in the area of health-related internet use would be considered. In this process, no disagreement was found. After coding was completed, authors synthesized the findings based on each research questions. The findings were then analyzed based on Eysenbach’s framework and the objectives of this review [32]. The perceptions of those participating in the various studies about health-related internet use and the prevalence of participants that had ever used the internet for health-related purposes were analyzed using descriptive statistics and the chi-square test to examine the differences between the studies published from 2007 to 2012 and 2013 to 2017.

## Results

### Characteristics of Study Participants

A total of 19 studies met the inclusion criteria, of which the majority (n=11) were conducted in the United States. Others were conducted in the United Kingdom, Canada, Israel, Nigeria, Sweden, and Uganda. In the selected studies, the majority of the adolescent participants were not suffering from any pertinent medical conditions (n=16); the remainder were identified as having juvenile arthritis [33], type 1 diabetes mellitus [34], or undergoing orthodontic treatment [35]. Apart from 2 studies whose sample specifically consisted of female [36] and male youths [37], all the studies included mixed gender. Hispanics made up the largest group in these studies, with proportions ranging from 9% [38] to 84% [39].

The types of communities people live in serves as a partial indicator of their socioeconomic status, and participants in the studies reported in the literature covered a wide range, from living in predominantly underserved, minority community areas [37,39-42] to middle class income areas [38] and urban areas [33,43-46]. The study settings, study participants, and their characteristics, along with their main findings are summarized in Multimedia Appendix 1. Three studies included participants who were incarcerated in a juvenile detention facility [40], who had run away from home and were homeless [47], and men who had sex with men (MSM) [37] to identify the characteristics of various subgroups of youth.

In total 10,974 participants took part in the selected studies, with those enrolled in individual studies ranging from as low as 24 to 6728. The average number of participants per study was 552. All the participants were 24 years or younger.

### Health-Related Internet Use

#### Prevalence of General Internet Use and Patterns of Health-Related Internet Use

The studies generally agreed that youths spend a large amount of time using the internet. According to the studies, 82.8% of youth in the age range of 11 to 18 years spend 1 to 4 hours/day online (Multimedia Appendix 2) [45] Interestingly, boys in the age range of 10 to 11 years reported using the internet for only 30 min/day, whereas youth in the age range of 14 to 15 years of both sexes were online for several hours/day [34]. Although the time youth in the age range of 16 to 17 years spent online drops to less than 1 hour/day [34], this is most likely related to the higher burden of academic work they are expected to accomplish at this age. Teens over 15 years reported more frequent use of the internet for searching health information than those younger than 15 years [41,42].

Researchers have also suggested that there is a difference in the frequency of daily internet usage in youths with sexual orientation differences. MSM youths exhibited significantly more frequent daily internet use (77%) than non-MSM (60%) when using it as a medium to search for their unique health information needs and to facilitate the development of their sexual identity [37]. Some of the venues used to access the internet were homes, schools, a friend’s home, an internet café, or the public library [48]; many runaway and homeless youth relied primarily on public access (ie, libraries and youth services agencies) [47].

A high frequency of internet use is also widely reported, and this finding is consistent across all the study countries. Sixty-four percent of youth (aged 10-16 years) in the United Kingdom are daily users, and a further 26% use the internet at least once or twice a week [35]. In the United States, 97% use the internet at least once a month, with 87% using it at least once a week [40]. For social networking, 87% maintain a personal social networking site (SNS) profile on MySpace or Facebook [41], and 96.6% use My Yearbook, Tagged, and Bebo [49]. Even in countries where access to the internet may be more limited, SNSs were popular with young people: in Nigeria, 73% reported that they had used the internet [46].

The findings of the various studies show that a high percentage of youth have used the internet for health-related purpose [33,37,39,41,48,50,51]. Among those (n=10) that reported ever using the internet for health-related purposes, the majority (80%, 8/10) found more than 50 percent have done so [33,39,41,47,48,50,51], whereas the remaining two reported fewer than 50% in the use of internet for this purpose [35,45]. There was no difference in the percentage of participants’ lifetime health-related internet use depending on the publication year when analyzed using a chi-square test (*P*>.05).

In a 2012 study of US teenagers, 81% reported that they had checked online health information, and 71% were very likely to search the internet for information on health; 59% sought online health information for their family’s health; and 56% had heard of Medline Plus [39]. In an earlier study of youth in the age range of 18 to 19 year, 65% reported the internet to be their primary source for health-related information [51]. However, this number was not consistent across populations and depended on specific conditions. A recent study in the United States found that 91.9% of youth with juvenile arthritis used the internet for more than 5 min/day, 69.4% used it for 30 min/day, and 36.6% for more than 1 hour/day [33]. Among youth undergoing orthodontic treatment in the United Kingdom, only 8% used the internet for specific disease-related information, and 3% had seen a phone app about orthodontics. Instead, their main source of information was their health care providers (HCPs), with only 8% using the internet as a primary source of information [35]. An Israeli study that compared Jewish and Arab middle and high school students’ internet access and health information–seeking behavior online found that although the two groups were similarly likely to access the internet, Arab students were far more likely to use the internet as a source of health information [48].

For the studies published from 2007 to 2012, daily users of the internet in this age group varied from 54.4% [47] to 88.2% [51], both in the United States. In studies published from 2013 to 2017, this had risen to from 64% [35] to 82.8% [45], both in the United Kingdom.

#### Device and Mode Used to Access the Internet

Although there has been a significant increase in the ownership of mobile phones by adolescents in recent years, many studies did not evaluate health information seeking via internet-enabled devices. Of those that did [35,40,41], the most common means for accessing the internet were personal computers or laptops (65%), followed by cell phones or other mobile internet-enabled devices (42%), with many reporting using both [40]. Stephens et al [35] asked specifically whether their study participants accessed mobile apps, and only 3% answered in the affirmative way, whereas in another study, one-third of the Native American youth reported that the use of their cell phone (36%) was a regular mode of internet access [41].

### Purpose of Health-Related Internet Use

Eysenbach’s framework indicates the major types of health-related internet use as consisting of information (content), support (community), communication, and electronic commerce (e-commerce) [32]. The findings of each study were therefore coded into three categories based on this framework; support and communication were combined into a single category for the purposes of this review. These are discussed in turn below.

#### Information

The primary purpose for health-related internet use is seeking information. The topics that young people search for online includes information on daily health-related issues [33-35,38,39,41,43,45-47,48], physical well-being [40,41,45,48,52], sexual health [33,37,42,45-47,48-51], mental health [33,41,44,52], social problems [33,34,36,37,44,52,50,51], and culturally and religiously sensitive topics [41,48]. Daily issues that play a significant role in young peoples’ lives, such as sports injuries, flu, chronic diseases, asthma, sexual health, fitness, and infections, are common areas of interest for youth on the internet [45]. This is particularly true for those suffering from particular diseases [33]. The internet also serves as a confidential source for information that may be culturally or religiously sensitive [48]; the greater likelihood of Arab youths seeking online information about mental health issues compared with their Jewish peers reflects the relative lack of mental health professionals available for Arab youth, as well as they being more culturally constrained than Jewish adolescents with regard to exposing personal concerns and problems [48]. On the other hand, there is some evidence to suggest that youth may be more likely to use the internet for less sensitive topics such as nutrition and exercise and less likely to look for sensitive topics such as violence, sexual health, bullying, tobacco, alcohol, drugs, and mental health [33]. Young people who are experiencing symptoms such as emotional difficulties often seek help for their feelings [52] and information related to their psychosocial health from peers online [44]. However, it has been reported that adolescents do not tend to use the internet for pain management [45]. Among those with diseases such as arthritis or diabetes, young people seek information related to their symptoms (52.4%) and treatment options (47.4%) [52] and may also turn to alternative sources (HCPs or peers) depending on the topic.

#### Support (Community) and Communication

Youths often use the internet to connect and create supportive communities on particular health issues, expressing interest in diverse online activities related to health, including messaging and connecting with others, networking, and receiving information. Intriguingly, 61.2% preferred an online support group to offline in-person groups [33], and children who were receiving hospital treatment in Sweden for a chronic disease, in this case diabetes, expressed a strong interest in using the internet for support networking, as well as for interpersonal contacts with their nondiabetic peers [34]. Youth with sexual orientation differences found the internet helpful as a way to connect to the gay community and meet partners online, as well as enabling them to discuss safe sex practices and boundaries and exchange information on HIV status before meeting prospective partners offline [37]. Interestingly, email communications with HCPs were not reported as a major purpose of health-related internet use.

#### Electronic Commerce

None of the studies included in this review examined young people’s health-related internet use for e-commerce.

### Factors Associated With Health-Related Internet Use

Gender, age, and in-school status are associated factors for the frequency of health-related internet use [34,45]. Girls tend to use the internet more often for help seeking online [41,45]. Youth of both sexes aged 16 to 17 years reported the internet to be their primary source for information, whereas those aged 10 to 11 years regarded their parents as their main source for information [34]. Similarly, youth aged 12 to 14 years regarded parents, teachers, and other adults as their primary source of health information, including sexual health [42]. Perhaps it may not be surprising that girls in Nigeria who are in school are more capable of finding information online than those who are out of school [36]. Only one study considered a potential association with race and ethnicity, reporting that among MSM in the United States, whites used the internet more frequently compared with African American and Latin American youths [37].

Notably, youths’ emotional characteristics and engagement in risky behaviors are associated with internet use [33]. Young people who have a lower psychosocial quality of life tended to have higher use of the internet for health-related matters [33], although there was no association with coping skills or pain frequency [45]. Additionally, youth who engage in high risk behaviors such as smoking, less physical activity, less sun protection activity, and depression were more willing to use technology for health promotion [38].

Electronic health (eHealth) literacy level was positively associated with seeking health information online [39], as were exposure to a health course, online information seeking, exposure to MedlinePlus, parents’ need for an interpreter when communicating with HCPs, upper grade in school, financial status higher health-related self-efficacy, and ethnicity (non-Hispanic), all of which are associated with a higher level of eHealth literacy [39]. An exposure to a specific website such as Medline online is known to facilitate health-related internet use; those enrolled on campuses promoting careers in the health care field and exposure to a health course are more likely to have heard of Medline Plus, and 11th graders are more likely to use Medline Plus than 9th or 10th graders [39]. Youth whose parents need interpreters to communicate between a family member and an HCP are also more likely to have heard of Medline Plus [39]. However, no association was found between access to technology and willingness to engage in eHealth literacy [38].

### Perceptions of Health-Related Internet Use

Overall, children and adolescents’ perception of health-related internet use is positive. The information presented in Table 1 for the quantitative studies includes whether the findings of each study indicate positive or negative perceptions of health-related internet use. The key evidence supporting this finding is also summarized. This perception is based on participants’ overall perceptions, the likelihood they will search online for health-related information, and participants’ trust, preference, and interest in using the internet as their primary source for health-related purposes. Among the studies that reported the participants’ perceptions on health-related internet use quantitatively (n=12), 50% (6/12) indicated that young people have generally positive perceptions about health-related internet use, with only 33.3% (4/12) reporting that children and adolescents have overall negative perceptions and 17% (2/12) reporting neutral perceptions. When we analyzed whether the perception depended on the publication year using a chi-square test, there was no statistical difference.

**Table 1:** Conceptual definitions and theoretical backgrounds.

Key concept related to health-related internet use	Definition and sources	Theoretical background	Authors and studies
eHealth^a^ literacy	“Ability to seek, find, understand, and appraise health information from electronic resources and apply such knowledge gained to addressing or solving health problem” [53]	—^b^	Manganello et al, 2016 [43]
Health information–seeking behavior	“Purposive search for health-related information to satisfy a query” [54]	—	Stephens et al, 2013 [35]
eHealth promotion	“Web-based health education and behavior change applications” [28]	Theory of planned behavior; problem behavior theory	Tercyak et al, 2009 [38]
eHealth intervention	“Integration of information and communication technology.”	—	Johnson et al, 2015 [33]
Electronic mental health	“Use of information and communication technologies to improve mental health.“ [55]	—	Wetterlin et al, 2014 [52]
Help seeking (help seeking online)	“Seeking assistance from mental health services, other formal services, or informal support sources for the purpose of resolving emotional or behavioral problem” [56]	Andersen behavioral model and Pescosolido’s network episode model	Barman-Adhikari et al, 2011 [47]
e-patient	“Those who bring information obtained online to the medical consultation” [57]	—	Neumark et al, 2013 [48]
None	—	—	Buhi et al, 2009 [51]; Fergie et al, 2013 [44]; Gaskin et al, 2012 [40]; Ghaddar et al, 2012 [39]; Henderson et al, 2013 [45]; Magee et al, 2012 [50]; Mustanski et al, 2011 [37]; Nordfeldt et al, 2013 [34]; Nwagwu, 2007 [36]; Rushing et al, 2011 [41]; Selkie et al, 2011 [49]

^a^eHealth: electronic health.

^b^Not provided.

#### Perceived Benefits

Regarding the perceived importance and usefulness of the internet, 90% of the participants in one study responded that having access to health-related resources on the Web is important [40], but only 8% of those in another study stated that their preferred source of information was the internet [35]. When adolescents are asked specifically about their sexual health–related use, 48.1% reported that they are relieved or comforted by the information online [52]. This positive perception is consistent with those found in a study on youth who have been detained in a juvenile detention facility, where 90% believed that access to information on various websites was useful [40]. However, young people have also reported that they would prefer sexual health Web-based sources to contain more comprehensive [50] or broader spectrum of topics [41] rather than just sexual health information.

User-generated content is perceived as advantageous for online health content as it provides diverse views and experiential knowledge combined with anonymity [44]. Many youth with sexual orientation differences reported that the internet facilitated the development of their sexual identity by connecting them with the gay community (both online and in real life), as well as by helping them search for specific facts about HIV or sexually transmitted infections (STIs), attempt self-diagnoses of symptoms they might be experiencing, find health centers that offer HIV or STI testing and affordable care, and learn about risk reduction techniques [37].

#### Perceived Barriers

In a Canadian study, 82.9% of the participants reported that they would be likely to use an information-based website at a difficult time in their life, but only 77% would be likely to use social media websites for information or to seek help [52]. The most commonly reported reason (62%-80%) for not seeking online health information was a preference for receiving information from a health professional, suggesting the use of the internet as a supplementary means rather than a replacement [48]. Only 10.9% accessed the health-related websites recommended by experts, and 10.6% sought help from social media for problems such as anxiety or depression [52].

Online privacy was a key issue for youth [34], with 87.7% stressing the importance of online privacy, which was particularly important for those with a specific health problem such as mental health issues [48]. Looking for sexual health information online was also closely linked to privacy issues as many youth felt reluctant to speak with an HCP about sensitive issues surrounding sexuality and instead use the internet to avoid embarrassment and overcome privacy issues [47]. On the other hand, lesbian, gay, bisexual, and transgender (LGBT) youth identified fear as an obstacle to online sexual health behaviors because of the perceived stigma resulting from being “caught” [50]. Although there are different perceptions in the various subgroups, 85% of the youths detained in a juvenile detention facility claimed not to be concerned about the privacy of their health information on the internet when on password-protected sites [40].

Another strong concern among youth who use the internet was the accuracy of the information [44]. When youth were asked specifically about their sexual health–related internet use, 44.4% reported that they were confused by the information they found, 25.9% were frustrated by the lack of information or an inability to find the information needed, whereas 18.5% were overwhelmed by the sheer amount of information available on the internet [51]. Some of the online experiences reported by adolescent males were not positive, with several recounting being distressed by finding information on the internet that either negatively portrayed homosexuality or described the victimization of LGBT people [37]. Those with low health literacy (28%) were more likely to rate the health information found on the internet as usually or often accurate compared with those with high health literacy (14%) [43]. Remarkably, study participants considered finding local information to be more difficult than finding general information online [49,52].

#### Important Features for Usability and Current Practice

Adolescents noted that they used different strategies to evaluate factual information and user-generated opinions on social media websites [44]. They highlighted the importance of the initial impression of a website and whether it made a serious and trustworthy impression on them; as they value integrity and anonymity, they were cautious about sharing their personal information [34]. Young people also stressed the importance of updating websites regularly to add value by including information such as current and recent events, facts and statistics (eg, verifiable information), as well as improving the technical aspects of websites by incorporating eye-catching design, high-quality visuals, and multimedia rather than text, although 51.9% said they never or hardly ever checked when a site was last updated or reviewed by a medical professional [51]. Furthermore, plainness (ie, clear content and layout) was another important feature that youth preferred [34]. Culturally, sexually, and religiously relevant health information targeted to specific populations, such as particular ethnic groups or sexual orientations, was preferred by minority youths and youths with sexual orientation differences [37,41,48,50]; they also preferred open access sites that did not require log-ins [34]. Regarding content, study participants wanted more information related to medications (92%), immunizations (90%), and STI test results (80%) [40].

These findings were consistent across studies examining a specific topical health (eg, sexual health) [49,51]. Regarding internet use related to sexual health, adolescents wanted sexual health education sites to be easily accessible, understandable, and user-friendly and the resources provided to be trustworthy-credible, confidential, and offered in a nonthreatening way [49]. Young people also wanted more information on specific topics and in-person resources such as local clinic resources, as these were reported as the most challenging for them to find [49,51].

When youth search for sexual health–related information, they used Google, Yahoo, and Ask most often as the first search engines [49,51], then followed sponsored links and the first three search results; another common strategy was to check for converging information across multiple websites [37]. Wikipedia and “WebMD” were the source they considered as providing the most credible sexual health information [37,49].

### Conceptualization

The key concepts for health-related internet use in the studies were eHealth literacy, health information–seeking behavior, eHealth promotion, eHealth interventions, as well as electronic mental health, health seeking, and electronic patient websites (Table 1). These concepts were all based on online activities related to information seeking and understanding or communication activities for health issues, problems, and health promotion. eHealth promotion and eHealth intervention provided more nuanced definitions related to Web-based interventions and education.

Conceptual definitions were provided in only a few studies, and of these, only a few utilized a theoretical framework. In Tercyak and colleagues’ study [38], the frameworks used were the theory of planned behavior and problem behavior theory, which explain the basis of the common mechanisms of multiple behavioral problems and provided frameworks that focused on individuals’ motivation for eHealth promotion associated with their behavior changes. When the media influence was studied, the uses and gratifications theory [46] was applied. This theory assumed that users choose a particular medium as an avenue to actively participate while being goal-directed, rather than as mere passive recipients. This theory also considered that the medium gratifies psychosocial needs. Another study used grounded theory [49] for its theory development.

### Methodological Evaluation

#### Study Design

A summary of the methodological evaluation conducted for this review is shown in Multimedia Appendix 3. All the studies in the table are descriptive, with the majority being cross-sectional studies; 26% (5/19) are correlational studies. In the studies included in this review, 58% (11/19) used a quantitative study design, whereas 16 % (3/19) used a qualitative study design, and 26% (5/19) used mixed or multiple methods. For the quantitative studies, the reported rates of use and access to the internet among the study participants, as well as any associated factors related to their internet use, are identified. Generally, the qualitative and mixed-methods studies explored how youths perceived the benefits and barriers of health-related internet use.

#### Study Sample

Less than half of the studies 47% (9/19) used convenience sampling [33,38,40,41,43,45,47,50,52]; the remaining studies used purposive sampling strategies [34,35,44,49] and random sampling across multiple sites [39,42,46,48], with 2 studies using both convenience and purposive sampling [37,51]. Of the 11 quantitative studies, only 4 used random sampling techniques [36,39,42,48]. Over half of the studies used multiple sites for sampling (58%) or used multiple resources, for example, by recruiting from both online and offline communities. No studies specifically indicated a sample size justification.

#### Data Collection and Analysis

Online surveys (26%) were the most common data collection technique [33,39,45,51,52]. Most of the qualitative studies used focus groups, although a few conducted semistructured interviews. Most studies used investigator-developed questionnaires to assess health-related internet use. This poses a number of potential issues related to the validity and reliability of their questionnaires compared with existing instruments. The most common analytic technique used was descriptive, which includes descriptive statistics, univariate analyses (*t* test and chi-square test), and multivariate analyses (linear regression, logistic regression, and analysis of variance). None of the quantitative studies indicated the statistical assumptions applied, and few explained how missing data were treated. For the survey studies, the data are self-reported, which inevitably introduces bias. The analytic approaches used were generally appropriate for the level of data and measurement. For the qualitative studies, thematic analysis, content analysis, and inductive descriptive analysis were commonly used.

## Discussion

### Summary of Findings and Comparison With Previous Work

This review of the most recent research in this area has deepened our understanding of how young people seek information from the internet and its related support systems for their health care. Adolescents spend a great deal of time on the internet, with the majority spending more than 2 hours every day. Although there are some inconsistencies regarding the amount of time and frequency of health-related use, depending on the population and disease concerned, most young people have used the internet for health-related purposes, and it represents their most frequent source of information.

Overall, youth are positive about using the internet to search for health-related information. As their most frequently used information source, the internet is commonly used for health-related information by both healthy and nonhealthy youth. Among healthy adolescents, this information includes sensitive topics such as sexual health and violence, as well as less sensitive topics such as exercise and nutrition. For those who have been diagnosed with a medical condition, the topics searched also include finding treatment options, seeking support, and networking with fellow sufferers, which is consistent with other populations [58]. Although we found a great deal of evidence to suggest that those with specific diseases use the internet to find friends [34], this may actually be related to the unique characteristics of youth who are comfortable meeting people online. Moreover, young people tend to prefer using support groups rather than attending in-person meetings and are not particularly bound to people with similar diagnoses. These characteristics are likely to be at least partly because of the perceived benefits of internet use, as many adolescents consider the internet to be a safe space where they can share sensitive information. Young people are interested in finding information from reliable sources such as HCPs or experts, as well as user-generated information from their peers who may have experienced the same issue. Members of this generation believe that it is helpful to learn diverse views on health topics [34].

Despite their high level of health-related internet use, several perceived challenges have been reported. To ensure useful Web-based health interventions or sites available for youth, credible resources and privacy are vital for successful outcomes. Young people generally evaluate a site’s credibility based upon its appearance, frequent citation, and the website’s domain name such as .com, .gov, or .org, but often there is no easy way to tell [49,51]. For example, privacy and confidentiality on an SNS may indicate a lack of online help or support services in mental health [52]. Additionally, researchers have found that some adolescents have experienced difficulties when searching for specific information such as local resources, despite their competency in finding general information. User-friendly features such as sites that do not require visitors to log in are suggested as another important element that enhances usability. It is also important for sites to have good readability and be well organized. Finding the most recently updated sites or checking a website’s creators are less common practices among teenagers and represent an area where education may be helpful. There is a general perception that there is a lack of useful, reliable resources for the specific information they need, such as particular disease-related information or health care topics for adolescents. Sites that can provide reliable information for youths need to be developed.

There are important findings related to the characteristics of various subgroups for health-related internet use. Youths whose parents or older relatives are not eHealth literate, have no internet access, have low health literacy, and need interpreters have a particularly high usage of the internet and are very likely to seek health information online for their family. Interestingly, young people who are in juvenile detention facilities worry less about privacy issues and are more willing to share information on the internet, whereas the opposite is true for MSM youths, who fear stigmatization if someone finds out their search history. There are different patterns of health-related internet use depending on age, with older youth becoming more frequent users of the internet to seek information on their health. Young people who have previously taken courses or received education on internet use designed to enhance their eHealth literacy level, for example, become more competent in their health-related internet use, especially when evaluating websites, suggests the need for more extensive health literacy education [53]. No gender differences were reported for health-related internet use, except for one study that indicated girls tend to be more frequent internet users than boys for issues related to pain management. In-school education also supported youth competency for health-related internet use. Youths who have a high risk of engaging in risky behaviors tend to use the internet more often than youths with lower risk for health-related internet use, which indicates a serious need for high quality content designed specifically for preventing behavioral issues to be developed. However, the most significant gap in the research in this area is that there were no studies of children younger than 10 years. This exclusion is source for further research.

### Limitations of This Review

Although this study followed evidence-based guidelines and adopted a systematic approach, chances of human error in coding are inevitable. We used a wide range of different search terms to identify relevant papers, including “health-related internet use,” “eHealth,” “internet use for health-related purpose,” “Web-based resource,” “health information seeking,” and “online resource,” combined with “child,” “adolescent,” “student,” “youth,” and “teen” in the databases searched; however, our choice of keywords may have resulted in missing relevant research studies eligible for inclusion. Although we used search engines most commonly used in the field of health, namely PubMed, CINAHL, and PsycINFO, this data-based selection many have created potential errors or missed relevant studies that should have been included. Furthermore, there is some potential for subjectivity in analyzing the findings, although 2 different coders carefully reviewed and coded each study independently and then discussed the results while double-checking each process. When the authors coded the methodological approaches used in each study, we tried not to assume a specific approach unless it was specifically stated in the study. For example, where no specific approach used for sampling is stated in the study, we coded these as using convenience sampling. This may have led to some potential errors regarding what the various authors actually did in their studies. Furthermore, the measures used in each study varied, and the study samples were heterogeneous, so we were unable to directly compare the outcomes for health-related internet use across all the studies examined for this review. Thus, we were not able to compare the findings based on regional differences among the samples, for example.

### Implications

Although this is an emerging field of study, there have been no previous studies systematically reviewing existing research exploring the health-related internet use of teenagers and young adults. As increasing number of internet-based interventions are being developed and applied specifically to address the needs of young people, it is important to understand the characteristics of health-related internet use among youth. Although the internet is both easily accessible and widely accepted by adolescents, the so-called “digital natives,” we have only a limited understanding of the patterns and characteristics of youth health-related internet use. This study therefore provides an important overview of the research findings to date related to patterns of youth health-related internet use. Although young people are generally frequent users of the internet for their health care and are positive about the practice, there remains a great need for education to support their competent and appropriate use of the internet. Additionally, there is a need for more reliable Web-based sources to be developed for this population. This study’s findings include a consideration of the associated factors for health-related internet use that have an effect on adolescents’ general health behaviors. A major gap identified in the review was the lack of a conceptual definition of the term “health-related internet use.” Furthermore, the majority of the studies published to date have not been based on a specific theoretical framework. In addition, this review identifies several limitations of the identified studies regarding methodological issues and provides suggestions for the further rigorous research required to design efficient and effective interventions for this hard-to-reach population. HCPs and policy makers should consider how best to integrate these needs into their current practices and policies.

### Recommendations for Future Research

Future research in this area needs to address several major gaps in the research, strengthen research methods, and contribute to appropriate theory development, as well as refining and conceptualizing eHealth practice and health-related internet use. The characteristics of various subpopulations need to be identified and compared with the characteristics of young people in general in this respect. In particular, internet use by younger adolescents and children who are younger than 10 years has not yet been studied. A closer examination of this younger demographic will give us a more accurate understanding of when children are first exposed to the internet and at what point its influence becomes seriously important. In this way, we will be able to identify appropriate “teachable moments” and the critical age at which to teach young people the skills they will need to become eHealth literate. Past studies have tended to focus primarily on cross-sectional studies, and it would be worthwhile to explore the longitudinal outcomes of health-related internet use. In future research in this area, studies with high-level analyses and rigorous research methods need to be conducted. For example, this review identified several studies that revealed important associated factors, and although most of the existing studies used convenience sampling, it is important for future research to utilize randomized sampling to yield more generalizable results that are applicable to wider populations. Multivariate analyses of the factors identified in the studies reviewed here will also yield valuable information, and standard measures for health-related internet use need to be developed that are based on a clear conceptual understanding and theoretical foundation. Furthermore, nearly all the selected studies suffered from limitations when representing the diverse populations of adolescents, including their gender, race and ethnicity, socioeconomic status, and regional status, although minority populations made up over half of the study participants overall.

### Implications for Health Promotion Practice

As youth are using the Web more frequently than ever before and will continue to, it is important to develop a better understanding of how they actually use the internet for health-related support and information. On the basis of in-depth understanding of youth practice, it is vital to provide health education that provides eHealth literacy skills for this population. Studies showed that youth who learned about Medline Plus are more likely higher users of the internet and more confident of using internet-based sources.

First, it is important to evaluate various online health information-seeking skills currently being taught to adolescent in schools and examine how best to help them develop the skills they will need to obtain, comprehend, and process health information, as well as online health care system information [43]. In health education for adolescents, it is necessary to include the internet as a basic component, given that so many already use the internet for their health-related needs or will do so in the near future. Studies indicate that those with low health literacy were more likely to rate the internet as usually or often accurate than those with high health literacy (28% low vs 14% high). As those with low health literacy were also more likely to use the internet daily, it is particularly important to support youth health literacy levels.

Health disparities exist, and the internet may even contribute to these, so it is important to allocate resources to the population most in need of this type of assistance, taking into account the differences identified between groups with different ethnicities reported in the research reviewed here. Internet access is one of the major factors for health-related internet use and eHealth literacy (urban vs rural). It is important to develop health education programs that focus on boosting eHealth literacy [43].

There is a great deal of room for improvement in the existing Web-based programs for teenagers and young adults. Many of the participants in the studies reviewed indicated a desire for more Web-based resources for health that are not subject to the limitations of existing websites. For example, a greater emphasis should be placed on developing an awareness of cultural values related to culturally and religiously sensitive health-related topics that may be more relevant to certain genders and youth populations, including taking into account the need to protect their privacy from parental monitoring by masking the nature of their health information-seeking [41,48], which would greatly enhance usability. Furthermore, as sensitive topics such as sexual information or mental health issues are often information that young people seek on the internet, it is important to provide reputable sources that will be accepted by the target population; more diverse content that is specifically tailored to the needs and characteristics of young people also needs to be developed. For example, people in this age group are particularly vulnerable for risky behaviors, and although they are interested in knowing more about prevention, there is a lack of good resources available to them.

## Appendix

Multimedia Appendix 1 Summary of included studies.

Multimedia Appendix 2 Summary of characteristics of health-related internet use.

Multimedia Appendix 3 Methodological evaluation of study quality.

## Abbreviations

CINAHL: Cumulative Index of Nursing and Allied Health Literature
e-commerce: electronic commerce
eHealth: electronic health
HCP: health care provider
LGBT: lesbian, gay, bisexual, and transgender
MSM: men who have sex with men
SNS: social networking sites
STI: sexually transmitted infection
