# Supplementary material for: Health-Related Internet Use by Children and Adolescents: Systematic Review
Source: J Med Internet Res. 2018 Apr 3;20(4):e120. doi: 10.2196/jmir.7731 (PMC5904452; doi:10.2196/jmir.7731)
Supplement: Multimedia Appendix 1 [file jmir_v20i4e120_app1.pdf]

Appendix 1. Summary of included studies.

First author, year	Country	Number of participants	Characteristics of adolescents			Perceptions about health-related Internet use (HRIU)	Evidence (key findings)	Ever use Internet for health (>50%)	Associated factors
			Age (years or graders)	Demographics	Medical conditions				
Manganello et al, 2016 [1]	United States	48	14-17 years	Mostly female (70%), 7th-9th grade (30%) and 10-12th grade (70%), African American adolescents living in an urban location	None	Negative	• 65% low health literacy group (compared with 35% having adequate health literacy group)<br>• Low health literacy group reported greater problems with understanding information they found on the Internet, requiring assistance (33% low vs 8% high) and more likely to rate health information online as accurate (28% low vs 14% high), suggesting there may be a lack of skills for evaluating credibility<br>• Associated factor: youth with low health literacy less likely to	-	Health literacy level

							prefer general Internet use and more likely to need help understating health information obtained on their own		
Johnson et al, 2015 [2]	United States	134	14-19 years	Mostly female (83,6%), Caucasian (81.3%), living with two parents in an urban location	Juvenile arthritis	Positive	• Youth with low psychosocial quality of life (PSQL) report more frequent use of SNSs than their peers with high PSQL; more frequent searching for information about sensitive health topics; and high interest in using additional online tools to address unmet needs and to connect with other teens with juvenile arthritis<br>• 74.5% prefer online support group than in-person support groups<br>• 91% interested in using a website that is just for teens with arthritis	Yes	PSQL
Wetterlin et al, 2014 [3]	Canada	521	17-24 years	Mostly female (76.6%), East or Southeast Asian (44%) or European or Caucasian (35.5%), living in British Columbia	None	Positive	• Likelihood of visiting Web-based mental health resources during a difficult time in life: 82.9% information-based website with mainly text, 76.8% social media websites	-	-

				(86%) and currently in school (87.3%)			• Likelihood of contacting a human within a Web-based mental health resource: 83.9% online professional (eg, therapist or coach)		
Fergie et al, 2013 [4]	United Kingdom	34	14-18 years	Mostly female (70.6%), school pupils or university students, living in an urban area	None	Not applicable (N/A) (qualitative)	• Perceived disadvantages and advantages of health content online are identified		
Henderson et al, 2013 [5]	United Kingdom	105	11-18 years	Male (58%) and female (42%), most had experienced some form of pain in the last 3 months (80%), living in an urban area	None	Negative	• 13.3%=HRIU related to pain management<br>• Although majority (82%) indicate between 1 to 4 hours/day of Internet use, seeking information online about their pain coping, information, and management is not primary strategy of a healthy adolescent population in pain	No	Gender
Nordfeldt et al, 2013 [6	Sweden	24	10-17 years	Male (54%) and female (46%), living in catchment area of a county hospital in the South-East of Sweden, either in compulsory or	Type 1 diabetes mellitus	N/A (qualitative)	• Primary source for information is parents or significant others	-	-

				secondary school, all had Internet access at home					
Neumark et al, 2013 [7]	Israel	6728	7th-12th grades	Male (48.9%) and female (51.1%), Jewish (71.8%) and Arab (28.2%) students	None	Neutral	• 52.1% reported having sought online health information in the past year<br>• Trust in online health information: high 15%, moderate 68.2%, low 16.8%<br>• Perceived Internet skill levels: high 37%, moderate 36.9%, low 26.1%<br>• Likelihood of HRIU: Arab students (63%), Jews (48%)<br>• Reasons for not seeking online health information: preference to receive information from a health professional, lack of interest, lack of trust, insufficient English proficiency, lack of time and privacy, limited access, expense, insufficient Internet skills	Yes	Age, frequency of Internet use, level of trust, Internet skills, medical condition, school performance
Stephens et al, 2013 [8]	United Kingdom	15 (interview) + 50 (survey)	10-16 years	Majority use social networking sites (SNSs) (76%)	Undergoing orthodontic treatment	Negative	• 8%=use Internet for orthodontic-related information<br>• Preferred source of information: health care providers (84%),	No	-

							peers (66%), parents (60%), leaflets (64%), Internet (8%)		
Gaskin et al, 2012 [9]	United States	79	13-18 years	Mostly male (84%), Hispanic (70%), youth detained in a juvenile detention facility, predominantly underserved, minority communities, high level of access to Internet	None	Positive	• 90% believe access to health information on website is useful<br>• 85% have no concerns about the privacy of their health information online on password-protected sites	-	-
Magee et al, 2012 [10]	United States	32	16-24 years	Male (53%), female (37.5%), and transgender (9.4%); African American (40.6%), Hispanic or Latino (25%), Caucasian (21.8%), Asian (3%), and multiracial (9%); lesbian, gay, bisexual, and transgender youth	None	Psitive	• 75% online searches for sexual health information as common activity<br>• Overall, seek facts and statistics about sexually transmitted infections (STIs) and human immunodeficiency virus (HIV) online (>70%) but few on the broader aspects of sexual health (<25%)	Yes	Fear of acquiring sexually transmitted disease
Ghaddar et al, 2012 [11]	United States	261	14-20 years	Mostly female (60%), Hispanic (84%), high school students; economically disadvantaged (58%)	None	Positive	• 81% had checked health information online<br>• 71% very likely to search the Internet for information on health	Yes	Health literacy

							• 59% sought health information for family's health online		
Selkie et al, 2011 [12]	United States	29	14-19 years	Mostly female (65.5%); heterosexual (61%); sexual experience (58.6%)	None	N/A (HRIU-qualitative)	• Common themes to the resources for sexual health information online are identified: accessibility, trustworthiness, andconfidentiality, and personal comfort	-	-
Barman-Adhikari et al, 2011 [13]	United States	169	13-24 years	Mostly male (68.2%), heterosexual (68.9%), gay or lesbian or bisexual (31.1%); African American (31%), Caucasian (26.9%), mixed race (18.5%), Hispanic or Latino (12.6%), and other (10.8%); runaway and homeless adolescents in an urban area	None	N/A	• 61% use Internet to find answers to general health questions<br>• <50% look for information about HIV or other STIs and 40% information about sex or sexuality	Yes	Gender, race or ethnicity, frequency of Internet use; personal access to computers

Mustanski et al, 2011 [14]	United States	329 (including 16 interviewees)	18-24 years	All male born, male identified, Caucasian (48%), African American (20%), Hispanic or Latino (20%), other (12%); men who have sex with men identified as gay (49%), bisexual (8%), queer (3%), heterosexual (39%)	None	N/A	• 88% used the Internet to find HIV or STI-related information	Yes	Race or ethnicity and gender
Rushing et al, 2011 [15]	United States	405	13-21 years	Male (43%) and female (57%), American Indian, American Native youth living in rural (58%) and urban (39%) communities	None	N/A	• 76% used the Internet to get information on a health topic	Yes	Gender and age
Buhi et al, 2009 [16]	United States	34	18-19 years	Mostly female (67.6%), 1st year under graduate students, Caucasian, reported as experienced Internet users	None	Positive	• 65% Internet is the predominant source for seeking health-related information<br>• 73.5% ever look online for information on specific diseases or medical problems, treatment, or procedures	Yes	-
Tercyak et al, 2009 [17]	United States	332	11-12 years	Mostly female (70%), African American (44%) or Caucasian (39%),	None	Neutral	• Moderate level of willingness to engage in electronic health (eHealth) promotion	-	-

				living in predominantly middle class income areas			• <20% are unwilling to engage in any eHealth promotion activity		
Ybarra et al, 2008 [18]	Uganda	500	12-18 years	Mostly male (61%), secondary school students (8th-11th grade) living in rural locations	None	Negative-	• Source of health information: parents or other adults (81%) and the Internet (38%)	-	-
Nwagwu, 2007 [19]	Nigeria	1145	13-19 years	All female, currently in school or dropped out of school, living in an urban area[a]	None	N/A	• Main source of health information in in-school girls: parents (66.2%) and teachers (56.2%)<br>• Main source of health information in out-of-school girls: friends (63.1%) and the Internet (55.2%)<br>• More out-of-school than in-school girls use the Internet to obtain information about their reproductive health	-	In-school or out-of-school status

Abbreviation: AA, African American; DM, diabetes mellitus; HIV, human immunodeficiency virus; HS, high school; LGBT, lesbian, gay, bisexual, and transgender; MSM, men who have sex with men; PS-QL, psychosocial quality of life; SNS, social networking sites; STI, sexually-transmitted infection; UG, undergraduate; UK, United Kingdom; USA, United States of America; yrs, years.

[a] The study states that participants were both educated and affluent.

References

1. Manganello JA, Sojka CJ. An exploratory study of health literacy and African American adolescents. Compr Child Adolesc Nurs 2016 Jul;39(3):221-239. doi: 10.1080/24694193.2016.1196264

2. Johnson KR, Fuchs E, Horvath KJ, Scal P. Distressed and looking for help: Internet intervention support for arthritis self-management. J Adolesc Health 2015 Feb;56(6):666-671. doi:10.1016/j.jadohealth.2015.02.019. PMID:26003583

3. Wetterlin FM, Mar MY, Neilson EK, Werker GR, Krausz M. eMental health experiences and expectations: a survey of youths' Web-based resource preferences in Canada. J Med Internet Res 2014 Dec;16(12):e293. doi:10.2196/jmir.3526. PMID:25519847

4. Fergie G, Hunt K, Hilton S. What young people want from health-related online resources: a focus group study. J Youth Stud 2013 Aug;16(5):579-596. doi:10.1080/13676261.2012.744811. PMID:24748849

5. Henderson EM, Keogh E, Rosser BA, Eccleston C. Searching the Internet for help with pain: adolescent search, coping, and medication behaviour. Br J Health Psychol 2013 Feb;18(1):218-232. doi:10.1111/bjhp.12005. PMID:23126577

6. Nordfeldt S, Angarne-Lindberg T, Nordwall M, Ekberg J, Bertero C. As facts and chats go online, what is important for adolescents with type 1 diabetes? PLoS One 2013 Jun 21;8(6):e67659. doi:10.1371/journal.pone.0067659. PMID:23805322

7. Neumark Y, Lopez-Quintero C, Feldman BS, Hirsch Allen AJ, Shtarkshall R. Online health information seeking among Jewish and Arab adolescents in Israel: results from a national school survey. J Health Commun 2013;18(9):1097-1115. doi: 10.1080/10810730.2013.778360. PMID: 23829662

8. Stephens R, Ryan FS, Cunningham SJ. Information-seeking behavior of adolescent orthodontic patients. Am J Orthod Dentofacial Orthop 2013 Mar;143(3):303-309. doi:10.1016/j.ajodo.2012.10.018. PMID:23452963

9. Gaskin GL, Longhurst CA, Anoshiravani A. Internet access and attitudes toward online personal health information among detained youth. Pediatrics 2012 Nov;130(5):914-17. doi:10.1542/peds.2012-1653. PMID:23090346

10. Magee JC, Bigelow L, Dehaan S, Mustanski BS. Sexual health information seeking online: a mixed-methods study among lesbian, gay, bisexual, and transgender young people. Health Educ Behav 2012 Jun;39(3):276-289. doi:10.1177/1090198111401384. PMID: 21490310

11. Ghaddar SF, Valerio MA, Garcia CM, Hansen L. Adolescent health literacy: the importance of credible sources for online health information. J Sch Health 2012 Jan;82(1):28-36. doi:10.1111/j.1746-1561.2011.00664.x. PMID:22142172

12. Selkie EM, Benson M, Moreno M. Adolescents' views regarding uses of social networking Websites and text messaging for adolescent sexual health education. Am J Health Educ 2011 Jul;42(4):205-12. PMID:22229150

13. Barman-Adhikari A, Rice E. Sexual health information seeking online among runaway and homeless youth. J Soc Social Work Res 2011 Jun;2(2):88-103. doi:10.5243/jsswr.2011.5. PMID: 22247795

14. Mustanski B, Lyons T, Garcia SC. Internet use and sexual health of young men who have sex with men: a mixed-methods study. Arch Sex Behav 2011;40(2):289-300. doi: 10.1007/s10508-009-9596-1. PMID: 20182787

15. Rushing SC, Stephens D. Use of media technologies by Native American teens and young adults in the Pacific Northwest: exploring their utility for designing culturally appropriate technology-based health interventions. J Prim Prev 2011;32(3-4):135-145. doi:10.1007/s10935-011-0242-z. PMID: 21805055

16. Buhi ER, Daley EM, Fuhrmann HJ, Smith SA. An observational study of how young people search for online sexual health information. J Am Coll Health 2009 Sep-Oct;58(2):101-11. doi:10.1080/07448480903221236. PMID:19892646

17. Tercyak KP, Abraham AA, Graham AL, Wilson LD, Walker LR. Association of multiple behavioral risk factors with adolescents' willingness to engage in eHealth promotion. J Pediatr Psychol 2009 Jun;34(5):457-469. doi:10.1093/jpepsy/jsn085. PMID:18723566

18. Ybarra ML, Emenyonu N, Nansera D, Kiwanuka J, Bangsberg DR. Health information seeking among Mbararan adolescents: results from the Uganda Media and You survey. Health Educ Res 2008 Apr;23(2):249-258. doi:10.1093/her/cym026. PMID: 17639121

19. Nwagwu WE. The Internet as a source of reproductive health information among adolescent girls in an urban city in Nigeria. BMC Public Health 2007 Dec;7(1):354. doi:10.1186/1471-2458-7-354. PMID:18096032
