# Supplementary material for: Health-Related Internet Use by Children and Adolescents: Systematic Review
Source: J Med Internet Res. 2018 Apr 3;20(4):e120. doi: 10.2196/jmir.7731 (PMC5904452; doi:10.2196/jmir.7731)
Supplement: Multimedia Appendix 2 [file jmir_v20i4e120_app2.pdf]

Appendix 2. Summary of characteristics of health-related internet use.

First author, year	General Internet use	Health-related Internet use		
	Prevalence	Prevalence	Purpose	Characteristics (Associated factors and perceptions)
Manganello et al, 2016 [1]	• 28% use a computer daily<br>• 77% use a computer a few times a week<br>• 37% did not have Internet access at home		• 7% use Internet often for health information<br>• 14% use Internet to get a question answered	• *Associated factors*: youth with low health literacy less likely to prefer general Internet use and more likely to need help understanding health information obtained on their own<br>• (low health literacy group [65%] > adequate health literacy group [35%]) 43%: Internet is the most helpful in providing health information among different media sources<br>• 58%: able to understand the health information they get from the Internet and media sources<br>• 33%: Internet is sometimes accurate<br>• 24%: Internet is usually or often accurate (those with low health literacy rated Internet more accurate than those with high health literacy)
Johnson et al, 2015 [2]	-	• 91.9% more than 5 min/day for health-related Internet use (HRIU)<br>• 69.4% more than 30 min/day for HRIU<br>• 36.6% more than 1 hour/day for HRIU	• Information on exercise (87.3%), nutrition (83.6%), and other health topics not related to arthritis (70.1%), mental health issues (50.7%), puberty (43.2%), sexual health (42.5%), drug use (27.6%), bullying (23.9%), and tobacco use (20.1%)<br>• Instant messaging or chatting with same disease (85.0%)<br>• Online forum (94.0%)	• *Associated factors*: low psychosocial quality of life<br>• 74.5%% prefer online support group to in-person support groups<br>• 91% interested in using a website that is just for teens with arthritis

			• Building personal profiles and networking (87.3%)	
Wetterlin et al, 2014 [3]	-	-	• Information (symptoms [52.4%], treatment options [47.4%], Web-based questionnaires or assessment tests [23.8%], prevalence rates [17.3%], peer support [13.1%], and other [2.5%])<br>• Seeking help for their feelings	• *Likelihood of visiting Web-based mental health resources during a difficult time in life*: 82.9% information-based website with mainly text, 76.8% social media websites<br>• *Likelihood of contacting human within a Web-based mental health resource*: 83.9% online professional (eg, therapist or coach)<br>• 87.7% reported that online privacy is very important<br>• 10.9% accessed to the recommended websites<br>• 10.6% used social media (eg, Facebook and MySpace) for help seeking with problems such as anxiety or depression<br>• *Important features*: description of interventions and treatments, evidence-based information, local resources, self-guided Web-based interventions, self-help information and tools, quizzes and tools to help asses mode and behavior, pictures and videos to help explain topics
Fergie et al, 2013 [4]	-	-	• Information (including psychosocial health information by peers)<br>• Support (connecting and creating supportive communities on particular health issues)	• *Perceived disadvantage of health content online*: likelihood of unreliable information<br>• *Perceived advantages for health-related content online*: relatable user-generated content (experiential knowledge), anonymity, diverse views<br>• Different strategies are used to evaluating factual and social media websites
Henderson et al, 2013 [5]	• 82.8%=1-4 hours/day	-	• Information (eg, sports injuries, medical information, flu strains, chronic illness, general	• 13.3%= HRIU related to pain management<br>• Facilitator: gender (girls > boys)

			health, asthma, sexual health, and fitness infections; 13.3%)	• No association related to coping skills or pain frequency
Nordfeldt et al, 2013 [6]	• 10-11 years: about 30 min/day<br>• 14-15 years: several hours/day<br>• 16-17 years, boys: less than 1 hour/day<br>• 16-17 years, girls: several hours/day	-	• Information<br>• Communicating and support (finding friends, including healthy youth<br>• Writing and sharing with others	• A majority of the participants had never visited the demonstrated websites before<br>• Aspects of security: expressed importance of impression, checking who's behind the website, sharing information cautiously (value with integrity and anonymity)<br>• Updating new value (current, recent events), facts (verifiable information), and eye-catching design to the sites is important<br>• Plainness (clear content and layout) is important<br>• Prefer open access sites
Neumark et al, 2013 [7]	• 97% of Jews: home access<br>• 89% of Arabs: home access<br>• Jewish: spent 17.9 hours/week<br>• Arab: 14.8 hours/week	• 52.1%=reported having sought online health information in the past year	• Information (most popular topic for search: both gender—fitness or exercise (67-81%); Girls—diet or nutrition or eating disorder, sexual function or menstruation or sexuality; Boys—contraception or pregnancy, alcohol or cigarettes or drugs)	• On average, 1 hour per week more online than those who did not seek health information online<br>• *Likelihood of HRIU*: Arab students (63%), Jews (48%)<br>• *Trust in online health information*: high 15%, moderate 68.2%, low 16.8%)<br>• *Perceived Internet skill levels*: high 37%, moderate 36.9%, low 26.1%<br>• *Associated factors*: grade (high school > middle school); level of trust in online health information, level of Internet skills, having discussed health or medical issues with a health care provider (HCP) in the past year, school performance, having consumed alcohol in the past year, and self-assessed health (in Arab students: those who rated their health as average are more likely to have HRIU than those in good health; No association with parental education)

				• *Reasons for not seeking online health information*: preference to receive information from a health professional, lack of interest, lack of trust, insufficient English proficiency, lack of time and privacy, limited access, expense, and insufficient Internet skills
Stephens et al, 2013 [8]	• 64% daily users<br>• 26% once or twice a week<br>• 92% use for homework<br>• 76% use for social networking	• 8% (use Internet for orthodontic-related information)<br>• 3% had seen a phone app about orthodontics	• Information	• *Main sources of information*: HCPs (84%), peers (66%), parents (60%), leaflets (64%), Internet (8%)<br>• *Preferences for sources of information*: orthodontist (84%), family (12%), general dentist (10%), digital versatile disc (10%), and readings leaflets (10%)
Gaskin et al, 2012 [9]	• 97% at least once a month<br>• 87% weekly use<br>• 65% most common means to use Internet is personal computer or laptops<br>• 42% access by cell phones or other mobile devices		• Information	• 90% believe access to health information on a website is useful<br>• Want access to medications (92%), immunizations (90%), and sexually transmitted infections (STIs) testing results (80%)<br>• Would share information with doctors (100%), parents (52%), or significant others (4%)<br>• 85% have no concerns about the privacy of their health information online on password-protected sites<br>• No association relayed to race
Magee et al, 2012 [10]	-	• 75% online searches for sexual health information as common activity	• Information about STIs or human immunodeficiency virus (HIV; 72%), preventive practices to reduce the risk of transmission of STIs (34%), specific agencies and locations for	• *Associated factor*: fear about being infected with STIs or HIV

			obtaining sexual health testing and/or treatment (22%), condoms or dental dams (19%), mechanics of sexual behaviors or pleasure (13%), relationship advice (13%), etc	• Prefer websites that offer comprehensive sexual health information (include preventive care, emotional and relationship elements)<br>• *Reasons for not accessing sexual health information online*: a sense of low personal relevance or disinterest (31%), fear of stigma (19%), mistrust of online information (16%)<br>• *Suggestions to improve online information*: website content, technical details, social connection, and lesbian, gay, bisexual, and transgender–specific suggestions
Ghaddar et al, 2012 [11]	-	• 81% had checked health information online<br>• 71% very likely to search the Internet for information on health<br>• 59% sought health information for family's health online	• Information	• 56% heard of Medline Plus (Facilitator: enrolled on campuses promoting careers in the health care field, 11th graders >9th or 10th, exposure to a health course, higher use of HRIU, need an interpreter to communicate between a family member and an HCP<br>• *Associated factors* for higher electronic health literacy level: exposure to a health course, online health information seeking, exposure to MedlinePlus, parents' need for interpreter to communicate with HCPs, upper grade, financial status higher health-related self-efficacy, and ethnicity (non-Hispanic)
Selkie et al, 2011 [12]	• 96.6% maintain a personal social networking site (SNS) profile (93.1% Facebook, 89.7% MySpace, 41.4%	-	• Information (Topics interested in: pregnancy prevention, sexually transmitted infection, and relationships)	• Want sexual health education to be easily accessible, understandable, and user-friendly<br>• Want sexual health resources to be trustworthy, credible, and confidential<br>• Want information offered in a nonthreatening way<br>• Use different sources (HCPs or youth) depending on topic<br>• Use Google to find answers

	MyYearbook, Tagged, and Bebo)			• Want more information on in-person resources and local clinics
Barman-Adhikari et al, 2011 [13]	• 54.4% daily user	• 40.7% ever looked online for sex information<br>• 47.3% ever looked online for HIV or STI information<br>• 23.3% ever looked online for HIV testing information	• Information (61%: general health information, 47%: information about HIV or other STIs, 40%: information about sex or sexuality, 23% locate HIV testing services	• *Associated factors*: daily Internet use, personal Internet access, receive email forwards about health-related information, connected with parents (more likely to seek HIV or STI information, HIV testing information, sex information, and general health information).<br>• *Associated factors*: gender (males > females: sexual information), race or ethnicity (AA<CA), frequency of HRIU; involvement in online social sites; personal Internet access (more likely to seek HIV or STI information)
Mustanski et al, 2011 [14]	• 77% daily user<br>• 15% at most multiweek Internet use	• 100% ever used the Internet to find health information<br>• 88% used the Internet to find HIV or STI-related information	• Information (finding sexual health information online: HIV or acquired immunodeficiency syndrome [AIDS]—88%, prevention, skills, and sexual health needs for men who have sex with men [MSM]),<br>• Support: minority identity development and self-acceptance (connecting to the gay community), sexual behavior with partners met online (facilitate safer sex discussion and screening)	• *Associated factors*: race or ethnicity (whites use Internet more often generally and for health-related purpose than blacks and Latinos after controlling for age and education) and gender (controlling for age, education, and ethnicity, gay and bisexual)<br>• men reported significantly more frequent Internet use for general use and health-related purpose)<br>• *Strategies to determine the accuracy of the information*: website "WebMD," cross checking across multiple websites and information provided on university of government website perceived as reliable
Rushing et al, 2011 [15]	• 73% 30 or more min per day (of which 25% 1-2 hours of use/day)<br>• 75% daily users	• 76% used the Internet to get information on a health topic<br>• 40% searched online for a sexual	• Information (diet, nutrition, exercise, or fitness [50%]; specific illnesses or medical conditions [47%; drugs or alcohol [42%]; sexual health, sexually transmitted diseases [STDs] or HIV [32%]; depression, anxiety, stress, or suicide [32%])	• *Associated factors*: gender (female > male); age (16-18 years > 13-15 years)<br>• Want a health and wellness website that contains a broad spectrum of topics such as physical fitness and exercise (57%), drug and alcohol use (50%), nutrition (46%), stress (42%), and traditional methods of

	• 50% home access<br>• 47% school<br>• 36% mobile<br>• SNS: having profiles: My Space or Facebook [87%])	health topic in the past		American Indian (AI) or American native (AN) healing (30%)<br>• Prefer accessing websites on sexual health information that contain broad spectrum of topic relevant to youth (ie, current events, health and wellness, social-relational issues, and academic topics) compared with those that only cover information on sexual health<br>• Pictures, videos, interactive "ask the experts" components, music or audio are attractive multimedia design features, and AI or AN relevant graphics, symbols, and designs are most comfortable features on health and wellness Web pages
Buhi et al, 2009 [16]	• 88.2% use "several times a day"	• 65% Internet is the predominant source for seeking health-related information<br>• *Topics for HRIU*: sexual health education (76.5%); specific diseases or medical problems (73.5%); medical treatments or procedures (73.5%); diet, nutrition, vitamin, or nutritional supplements (70.6%); depression,	• *Information (topics that they have used the Internet for*: STI including HIV (70.6%), male or female genital (58.8%), preventing pregnancy (52.9%), contraceptives (50%), normal sexual behavior (44.1%), abortion (44.1%), prescription drugs and sexual health (41.2%), alcohol and/or illicit drug use and sexual health (41.2%), drug-facilitated rape (38.2%), and sexual assault and/or rape (35.3%))	• 35.5% sexual information from a physician<br>• 32.4% sexual information from relatives<br>• 32.4% sexual information from friends<br>• 63% reassured they could make appropriate health care decisions<br>• 48.1% relieved or comforted by the information online<br>• 44.4% confused by the information<br>• 33.3% eager to share the information<br>• 29.6% confident in raising new questions or concerns<br>• 25.9% frustrated by lack of information or inability to find information needed<br>• 18.5% overwhelmed by the amount of the information<br>• 51.9% never or hardly ever check the date of the last update or review by a medical professional<br>• *Search engines*: Google (94.1%), Yahoo, and Ask<br>• 26.5% followed "sponsored link"<br>• 79.3% followed the first three search results

		anxiety, stress, or mental health issues (64.7%); environmental health hazards (55.9%); problems with drugs or alcohol (47.1%); experimental treatments or medicines (47.1%); alternative treatments or medicine (47.1%); particular doctor or hospital (44.1%); and Medicare or Medicaid (20.6%)		• Most challenging search topic identified as locational information for specific local clinics<br>• Wikipedia: source of credible sexual health information
Tercyak et al, 2009 [17]	• High level of access to technology (>95%)			• Moderate level of willingness to engage in electronic health (eHealth) promotion<br>• <20% showed no willingness to engage in any eHealth promotion activity<br>• Multiple behavioral risk factors (smoking, physical activity, sun protection, and depression) associated with willingness to use technology for health promotion<br>• No association between access to technology and willingness for eHealth literacy engagement
Ybarra et al, 2008 [18]	-	-	• Information (related to health and disease, HIV or AIDS, and sexual health)	• *Associated factors*: age (15-18 years > 12-14 years)

				• *Sources of health information*: parents or other adults (81%), Internet (38%)<br>• When asked if the Internet was free to use, HIV or AIDS (66%), alcohol and drug issues (24%), and depression and suicide (16%) would be searched
Nwagwu, 2007 [19]	• 73.1%=ever used the Internet (Home access: in school (43.9%), out of school (5.6%)		• Information (topics included STD, HIV or AIDS, diet or nutrition, fitness or exercise, sexual activities, pregnancy, drug abuse, and sexual abuse)	• *Associated factors*: Inschool or out of school (In-school youth are more capable of finding information online)<br>• *Main source of health information for in-school girls*: parents (66.2%) and teachers (56.2%)<br>• *Main source of health information for out-of-school girls*: friends (63.1%) and the Internet (55.2%)<br>• More out-of-school than in-school girls use the Internet to obtain information about their reproductive health<br>• *Main reasons for HRIU*: privacy, relative information, unrestricted access, lack of alternatives, variety of information, and ease of access
Abbreviations: AIDS, acquired immunodeficiency syndrome; d, day; HIV, human immunodeficiency virus; HRIU, health-related Internet use; hr, hour; hrs, hours; LGBT, lesbian, gay, bisexual, and transgender; min, minutes; mo, month; r/t, related to; MSM, men who have sex with men; SNS, social networking sites; STD, sexually transmitted disease; STI, sexually transmitted infections; wk, week; yrs, years.				

References

1. Manganello JA, Sojka CJ. An exploratory study of health literacy and African American adolescents. Compr Child Adolesc Nurs 2016 Jul;39(3):221-239. doi: 10.1080/24694193.2016.1196264

2. Johnson KR, Fuchs E, Horvath KJ, Scal P. Distressed and looking for help: Internet intervention support for arthritis self-management. J Adolesc Health 2015 Feb;56(6):666-671. doi:10.1016/j.jadohealth.2015.02.019. PMID:26003583

3. Wetterlin FM, Mar MY, Neilson EK, Werker GR, Krausz M. eMental health experiences and expectations: a survey of youths' Web-based resource preferences in Canada. J Med Internet Res 2014 Dec;16(12):e293. doi:10.2196/jmir.3526. PMID:25519847

4. Fergie G, Hunt K, Hilton S. What young people want from health-related online resources: a focus group study. J Youth Stud 2013 Aug;16(5):579-596. doi:10.1080/13676261.2012.744811. PMID:24748849

5. Henderson EM, Keogh E, Rosser BA, Eccleston C. Searching the Internet for help with pain: adolescent search, coping, and medication behaviour. Br J Health Psychol 2013 Feb;18(1):218-232. doi:10.1111/bjhp.12005. PMID:23126577

6. Nordfeldt S, Angarne-Lindberg T, Nordwall M, Ekberg J, Bertero C. As facts and chats go online, what is important for adolescents with type 1 diabetes? PLoS One 2013 Jun 21;8(6):e67659. doi:10.1371/journal.pone.0067659. PMID:23805322

7. Neumark Y, Lopez-Quintero C, Feldman BS, Hirsch Allen AJ, Shtarkshall R. Online health information seeking among Jewish and Arab adolescents in Israel: results from a national school survey. J Health Commun 2013;18(9):1097-1115. doi: 10.1080/10810730.2013.778360. PMID: 23829662

8. Stephens R, Ryan FS, Cunningham SJ. Information-seeking behavior of adolescent orthodontic patients. Am J Orthod Dentofacial Orthop 2013 Mar;143(3):303-309. doi:10.1016/j.ajodo.2012.10.018. PMID:23452963

9. Gaskin GL, Longhurst CA, Anoshiravani A. Internet access and attitudes toward online personal health information among detained youth. Pediatrics 2012 Nov;130(5):914-17. doi:10.1542/peds.2012-1653. PMID:23090346

10. Magee JC, Bigelow L, Dehaan S, Mustanski BS. Sexual health information seeking online: a mixed-methods study among lesbian, gay, bisexual, and transgender young people. Health Educ Behav 2012 Jun;39(3):276-289. doi:10.1177/1090198111401384. PMID: 21490310

11. Ghaddar SF, Valerio MA, Garcia CM, Hansen L. Adolescent health literacy: the importance of credible sources for online health information. J Sch Health 2012 Jan;82(1):28-36. doi:10.1111/j.1746-1561.2011.00664.x. PMID:22142172

12. Selkie EM, Benson M, Moreno M. Adolescents' views regarding uses of social networking Websites and text messaging for adolescent sexual health education. Am J Health Educ 2011 Jul;42(4):205-12. PMID:22229150

13. Barman-Adhikari A, Rice E. Sexual health information seeking online among runaway and homeless youth. J Soc Social Work Res 2011 Jun;2(2):88-103. doi:10.5243/jsswr.2011.5. PMID: 22247795

14. Mustanski B, Lyons T, Garcia SC. Internet use and sexual health of young men who have sex with men: a mixed-methods study. Arch Sex Behav 2011;40(2):289-300. doi: 10.1007/s10508-009-9596-1. PMID: 20182787

15. Rushing SC, Stephens D. Use of media technologies by Native American teens and young adults in the Pacific Northwest: exploring their utility for designing culturally appropriate technology-based health interventions. J Prim Prev 2011;32(3-4):135-145. doi:10.1007/s10935-011-0242-z. PMID: 21805055

16. Buhi ER, Daley EM, Fuhrmann HJ, Smith SA. An observational study of how young people search for online sexual health information. J Am Coll Health 2009 Sep-Oct;58(2):101-11. doi:10.1080/07448480903221236. PMID:19892646

17. Tercyak KP, Abraham AA, Graham AL, Wilson LD, Walker LR. Association of multiple behavioral risk factors with adolescents' willingness to engage in eHealth promotion. J Pediatr Psychol 2009 Jun;34(5):457-469. doi:10.1093/jpepsy/jsn085. PMID:18723566

18. Ybarra ML, Emenyonu N, Nansera D, Kiwanuka J, Bangsberg DR. Health information seeking among Mbararan adolescents: results from the Uganda Media and You survey. Health Educ Res 2008 Apr;23(2):249-258. doi:10.1093/her/cym026. PMID: 17639121

19. Nwagwu WE. The Internet as a source of reproductive health information among adolescent girls in an urban city in Nigeria. BMC Public Health 2007 Dec;7(1):354. doi:10.1186/1471-2458-7-354. PMID:18096032
