# Supplementary material for: Health-Related Internet Use by Children and Adolescents: Systematic Review
Source: J Med Internet Res. 2018 Apr 3;20(4):e120. doi: 10.2196/jmir.7731 (PMC5904452; doi:10.2196/jmir.7731)
Supplement: Multimedia Appendix 3 [file jmir_v20i4e120_app3.pdf]

Appendix 3. Methodological evaluation of study quality

| First author, year | Research design | Conceptual framework | Sampling | Data collection method and instrument | Analysis method | Threats to validity |
|---|---|---|---|---|---|---|
| Manganello et al, 2016 [1] | Qualitative and Quantitative | None | Convenience sampling (community organizations) | Semistructured interviews | Semiquantitative analysis | Self-report; Limited generalizability |
| Johnson et al, 2015 [2] | Quantitative; Cross-sectional | None | Convenience sampling across multiple sites (clinics, advocacy organizations' websites, and Facebook) | Online survey using MyRheum, Illness Intrusiveness Scale, and investigator-developed questionnaire | Descriptive statistics, *t* test, chi-square test | Self-report and self-selection bias; heterogeneous sample |
| Wetterlin et al, 2014 [3] | Quantitative; Cross-sectional | None | Convenience sampling across multiple sites (Facebook and Twitter, online university student communities, and community organizations) | Online survey using Bell Youth Impact Survey and investigator-developed questionnaires | Descriptive statistics and descriptive analysis | Self-report and self-selection bias; Limited generalizability |
| Fergie et al, 2013 [4] | Qualitative | None | Purposive and snowball sampling across multiple sites (university course conveners and community youth group leaders) | Focus groups | Thematic analysis using a constant comparison method | Self-selection bias; Limited generalizability (homogenous sample) |
| Henderson et al, 2013 [5] | Quantitative; Cross-sectional; Correlational | None | Convenience sampling across multiple sites (four schools) | Online survey using investigator-developed questionnaires, pain coping questionnaire, etc | Descriptive statistics; chi-square test, correlational analyses (Pearson *r* test) | Self-report; Limited generalizability |
| Nordfeldt et al, 2013 [6] | Qualitative | None | Purposive sampling (catchment area of a county hospital) | Focus groups | Qualitative inductive analysis; content analysis | Self-selection bias; Limited generalizability |
| Neumark et al, 2013 [7] | Quantitative Cross- | None | Stratified sampling across multiple sites (five schools) | Survey | Multivariate regression | Self-report |

| | | | | | | |
|---|---|---|---|---|---|---|
| | sectional; Correlational | | | | | |
| Stephens et al, 2013 [8] | Qualitative and quantitative | None | Purposive sampling at a single site (hospital) | Semistructured interviews; Survey using investigator-developed questionnaire | Content analysis (framework approach) | Self-report and self-selection bias; Limited generalizability |
| Gaskin et al, 2012 [9] | Quantitative; Cross-sectional | None | Convenience sampling at a single site (juvenile detention facility) | Interviews using an investigator-developed questionnaire | Descriptive statistics | Self-report and self-selection bias; Limited generalizability because of single site |
| Magee et al, 2012 [10] | Mixed methods; Cross-sectional | None | Convenience sampling across multiple sites | Interviews | Descriptive thematic analysis (inductive approach) | Limited generalizability |
| Ghaddar et al, 2012 [11] | Quantitative; Cross-sectional; Correlational | None | Random sampling across multiple sites (four schools) | Online survey using investigator-developed questionnaires, General Self-Efficacy Scale, eHealth (electronic health) Literacy Scale, Newest Vital Sign | Descriptive statistics, Univariate tests (chi-square and *t* tests); multivariate analyses (linear and logistic regressions) | Self-report; Limited generalizability (single state) |
| Selkie et al, 2011 [12] | Mixed methods; Cross-sectional | Grounded theory | Purposive sampling across multiple sites (community centers and schools) | Focus groups; paper survey using Youth Risk Behavior Survey, the National Health and Nutrition Examination Survey | Descriptive analysis; Constant comparative method | Self-report and self-selection bias; Limited generalizability |
| Barman-Adhikari et al, 2011 [13] | Quantitative; Cross-sectional | Conceptual model for online sexual health information seeking among runways and | Convenience sampling at a single site (drop-in agency) | Survey | Logistic regression | Self-report; Limited generalizability |

| | | | | | | |
|---|---|---|---|---|---|---|
| | | homeless youths (based on Andersen behavioral model and Pescosolido's network episode model) | | | | |
| Mustanski et al, 2011 [14] | Mixed methods; Cross-sectional | None | Convenience and purposive sampling across multiple sites | Survey; Interviews | Descriptive analysis (inductive approach) | Self-report; Limited generalizability |
| Rushing et al, 2011 [15] | Quantitative; Cross-sectional | American Indian/ American Native Health Disparities and Wellness Model | Convenience sampling | Survey | Descriptive statistics; Chi-square analysis | Self-report; Limited generalizability |
| Buhi et al, 2009 [16] | Quantitative and qualitative; Cross-sectional | None | Convenience and purposive sampling at a single site (college) | Online survey using investigator-developed questionnaires; Observation (verbal report using "talking out loud" method) | Descriptive statistics; descriptive analysis | Self-report; limited generalizability because of single site |
| Tercyak et al, 2009 [17] | Quantitative; Correlational | Problem behavior theory; theory of planned behavior | Convenience sampling at a single site (hospital) | Survey; self-report using Adolescent eHealth Promotion Scale, National Centers for Disease Control and Prevention's Youth Risk Behavior Survey, Center for Epidemiologic Studies-Depression Scale | Multivariate linear regression | Self-report and self-selection bias; limited generalizability |
| Ybarra et al, 2008 [18] | Quantitative; Cross-sectional | None | Random sampling across multiple sites (five schools) | Survey | Logistic regression | Self-report |

| | | | | | | |
|---|---|---|---|---|---|---|
| Nwagwu, 2007 [19] | Quantitative; Cross-sectional; Correlational | Uses and gratifications theory | Random sampling across multiple sites (22 schools and community organizations) | Survey; Investigator-modified questionnaire | Descriptive statistics, chi-square and *t* tests; Regression analysis | Limited generalizability because of incomparability of proportions of in-school and out-of-school samples |
| Abbreviations: AI/AN, American Indian, American Native | | | | | | |

References

1. Manganello JA, Sojka CJ. An exploratory study of health literacy and African American adolescents. Compr Child Adolesc Nurs 2016 Jul;39(3):221-239. doi: 10.1080/24694193.2016.1196264

2. Johnson KR, Fuchs E, Horvath KJ, Scal P. Distressed and looking for help: Internet intervention support for arthritis self-management. J Adolesc Health 2015 Feb;56(6):666-671. doi:10.1016/j.jadohealth.2015.02.019. PMID:26003583

3. Wetterlin FM, Mar MY, Neilson EK, Werker GR, Krausz M. eMental health experiences and expectations: a survey of youths' Web-based resource preferences in Canada. J Med Internet Res 2014 Dec;16(12):e293. doi:10.2196/jmir.3526. PMID:25519847

4. Fergie G, Hunt K, Hilton S. What young people want from health-related online resources: a focus group study. J Youth Stud 2013 Aug;16(5):579-596. doi:10.1080/13676261.2012.744811. PMID:24748849

5. Henderson EM, Keogh E, Rosser BA, Eccleston C. Searching the Internet for help with pain: adolescent search, coping, and medication behaviour. Br J Health Psychol 2013 Feb;18(1):218-232. doi:10.1111/bjhp.12005. PMID:23126577

6. Nordfeldt S, Angarne-Lindberg T, Nordwall M, Ekberg J, Bertero C. As facts and chats go online, what is important for adolescents with type 1 diabetes? PLoS One 2013 Jun 21;8(6):e67659. doi:10.1371/journal.pone.0067659. PMID:23805322

7. Neumark Y, Lopez-Quintero C, Feldman BS, Hirsch Allen AJ, Shtarkshall R. Online health information seeking among Jewish and Arab adolescents in Israel: results from a national school survey. J Health Commun 2013;18(9):1097-1115. doi: 10.1080/10810730.2013.778360. PMID: 23829662

8. Stephens R, Ryan FS, Cunningham SJ. Information-seeking behavior of adolescent orthodontic patients. Am J Orthod Dentofacial Orthop 2013 Mar;143(3):303-309. doi:10.1016/j.ajodo.2012.10.018. PMID:23452963

9. Gaskin GL, Longhurst CA, Anoshiravani A. Internet access and attitudes toward online personal health information among detained youth. Pediatrics 2012 Nov;130(5):914-17. doi:10.1542/peds.2012-1653. PMID:23090346

10. Magee JC, Bigelow L, Dehaan S, Mustanski BS. Sexual health information seeking online: a mixed-methods study among lesbian, gay, bisexual, and transgender young people. Health Educ Behav 2012 Jun;39(3):276-289. doi:10.1177/1090198111401384. PMID: 21490310

11. Ghaddar SF, Valerio MA, Garcia CM, Hansen L. Adolescent health literacy: the importance of credible sources for online health information. J Sch Health 2012 Jan;82(1):28-36. doi:10.1111/j.1746-1561.2011.00664.x. PMID:22142172

12. Selkie EM, Benson M, Moreno M. Adolescents' views regarding uses of social networking Websites and text messaging for adolescent sexual health education. Am J Health Educ 2011 Jul;42(4):205-12. PMID:22229150

13. Barman-Adhikari A, Rice E. Sexual health information seeking online among runaway and homeless youth. J Soc Social Work Res 2011 Jun;2(2):88-103. doi:10.5243/jsswr.2011.5. PMID: 22247795

14. Mustanski B, Lyons T, Garcia SC. Internet use and sexual health of young men who have sex with men: a mixed-methods study. Arch Sex Behav 2011;40(2):289-300. doi: 10.1007/s10508-009-9596-1. PMID: 20182787

15. Rushing SC, Stephens D. Use of media technologies by Native American teens and young adults in the Pacific Northwest: exploring their utility for designing culturally appropriate technology-based health interventions. J Prim Prev 2011;32(3-4):135-145. doi:10.1007/s10935-011-0242-z. PMID: 21805055

16. Buhi ER, Daley EM, Fuhrmann HJ, Smith SA. An observational study of how young people search for online sexual health information. J Am Coll Health 2009 Sep-Oct;58(2):101-11. doi:10.1080/07448480903221236. PMID:19892646

17. Tercyak KP, Abraham AA, Graham AL, Wilson LD, Walker LR. Association of multiple behavioral risk factors with adolescents' willingness to engage in eHealth promotion. J Pediatr Psychol 2009 Jun;34(5):457-469. doi:10.1093/jpepsy/jsn085. PMID:18723566

18. Ybarra ML, Emenyonu N, Nansera D, Kiwanuka J, Bangsberg DR. Health information seeking among Mbararan adolescents: results from the Uganda Media and You survey. Health Educ Res 2008 Apr;23(2):249-258. doi:10.1093/her/cym026. PMID: 17639121

19. Nwagwu WE. The Internet as a source of reproductive health information among adolescent girls in an urban city in Nigeria. BMC Public Health 2007 Dec;7(1):354. doi:10.1186/1471-2458-7-354. PMID:18096032
